# Molar incisor hypomineralisation in relation to fat-soluble vitamin status and intestinal permeability in children with celiac disease

**DOI:** 10.3389/fnut.2026.1864281

**Published:** 2026-08-06

**Authors:** Lucian-Ioan Cristun, Raluca-Nicoleta Vasilescu, Niculina Mang, Iulius Jugănaru, Otilia Mărginean, Andrei-Ioan Munteanu, Delia-Maria Nicoară, Andrei Păun, Alexandra-Diana Cheleș, Laura Cristina Rusu

**Affiliations:** 1 Doctoral School, Victor Babeș University of Medicine and Pharmacy, Timișoara, Romania; 2 Multidisciplinary Center of Research, Evaluation, Diagnosis and Therapies in Oral Medicine, Victor Babeș University of Medicine and Pharmacy, Timișoara, Romania; 3 Louis Țurcanu Emergency Clinical Hospital for Children, Timișoara, Romania; 4 First Pediatric Clinic, Victor Babeș University of Medicine and Pharmacy, Timișoara, Romania; 5 Research Center for Growth and Development Disorders in Children (CREDE), Timișoara, Romania; 6 Clinic of Oral Pathology, Victor Babeș University of Medicine and Pharmacy, Timișoara, Romania

**Keywords:** celiac disease, enamel defect, molar incisor hypomineralisation, paediatric dentistry, vitamin D, vitamin K2, zonulin

## Abstract

**Background:**

Celiac disease (CD) is a systemic autoimmune enteropathy triggered by gluten ingestion that frequently manifests with extra-intestinal complications, including enamel defects. Molar incisor hypomineralisation (MIH) has been proposed as a dental marker of systemic mineral dysregulation; however, its association with fat-soluble vitamin deficiencies in children with celiac disease remains insufficiently characterised.

**Methods:**

A prospective pilot case–control study was conducted over 12 months including 70 children (35 with confirmed CD, 35 healthy controls). MIH severity was graded using a validated four-point scale; enamel defect extent was scored using the Aine classification. Serum levels of vitamins K1, K2, and D, zonulin, calcium, phosphorus, alkaline phosphatase, and oral hygiene indices (Löe Plaque Index; OHI-S) were recorded. An exhaustive statistical analysis was conducted using these parameters.

**Results:**

MIH was detected in 27/35 CD children (77.1%) versus 0/35 controls. Vitamin K2, vitamin D and serum calcium were significantly lower in CD. Strong negative correlations were observed between MIH severity, vitamin K2 and vitamin D. ROC analysis revealed excellent diagnostic performance of zonulin, vitamin K2, and vitamin D for discriminating CD status.

**Conclusion:**

Children with celiac disease exhibited a higher prevalence and greater severity of molar-incisor hypomineralisation, which was associated with lower vitamin K2 and vitamin D levels. Zonulin, vitamin K2, and vitamin D also showed promising discriminatory ability for identifying children with celiac disease; however, given the pilot nature of the study, these findings should be considered exploratory and require validation in larger multicenter cohorts.

## 1 Introduction

Celiac disease (CD) is a chronic immune-mediated enteropathy affecting approximately 1–2% of the global population, triggered in genetically susceptible individuals by dietary gluten (1, 2). The clinical spectrum of CD is broad, encompassing classical gastrointestinal symptoms — chronic diarrhoea, malabsorption, and failure to thrive, as well as a growing array of extra-intestinal manifestations, amongst which dental enamel defects hold particular diagnostic significance (3).

Dental enamel hypoplasia and hypomineralisation in CD were first systematically described by Aine et al. in 1990, who observed that affected children displayed bilaterally symmetric enamel lesions that did not correspond to the classical pattern of local traumatic or infectious injury. These defects, now recognised as an independent diagnostic criterion for CD, are caused by the systemic metabolic disturbances, principally malabsorption of calcium, phosphorus, vitamin D, and vitamin K, that arise during the critical period of enamel mineralisation (4, 5). Vitamin D deficiency secondary to fat-soluble vitamin malabsorption and villous atrophy may further aggravate hypocalcaemia in celiac disease by impairing active intestinal calcium absorption and contributing to secondary hyperparathyroidism. This mechanism has been described as an important contributor to metabolic bone disease in CD (6, 7). Consequently, calcium malabsorption in celiac disease is considered multifactorial, involving steatorrhoea, impaired vitamin D-dependent calcium transport, and reduced vitamin D availability (8, 9). Additional disturbances in phosphorus homeostasis and vitamin K-dependent mineralisation pathways have also been proposed as potential modulators of enamel mineralisation, although their independent contribution to dental enamel defects in celiac disease remains incompletely defined (10, 11).

Molar incisor hypomineralisation (MIH) is a qualitative developmental enamel defect characterised by demarcated opacities affecting the first permanent molars, with or without involvement of the permanent incisors (12). Its aetiology is multifactorial, encompassing prenatal, perinatal, and postnatal systemic disruptions of ameloblast function (13). The association between MIH and CD has attracted increasing attention, as the systemic nutrient deficits inherent to CD, particularly of fat-soluble vitamins, may compromise the protein-mediated mineralisation processes essential for enamel matrix maturation (14). MIH has been shown to be most prevalent amongst newly diagnosed and non-compliant children with CD, who also exhibit significantly lower vitamin D levels and elevated tissue transglutaminase values, suggesting that the severity of intestinal disease, rather than diagnosis alone, modulates enamel defect expression (15).

Amongst the fat-soluble vitamins, vitamin K2 (menaquinone) plays a pivotal role in the carboxylation of osteocalcin and matrix Gla protein (MGP), both of which are essential for calcium deposition in mineralised tissues (16). Vitamin D, acting synergistically with vitamin K2, regulates intestinal calcium absorption and osteoblast function (17). In CD, the villous atrophy-mediated malabsorption of these vitamins is well documented, yet their specific contribution to enamel defect severity in children with MIH has not been comprehensively investigated (18).

A critical, yet underexplored, upstream determinant of the micronutrient malabsorption underlying these defects is the integrity of the intestinal mucosal barrier. Zonulin, a modulator of tight-junction permeability, is elevated in CD and serves as a serum biomarker of intestinal dysbiosis and mucosal permeability (12). Its role in paediatric CD diagnosis and its correlation with oral manifestations have not been previously explored in a structured case–control setting.

While previous studies have mainly focused on the prevalence of enamel defects in children with celiac disease, relatively little attention has been given to the potential relationship between enamel mineralisation and biomarkers reflecting intestinal permeability and micronutrient status. Characterising these associations is not only of pathophysiological interest but may inform non-invasive diagnostic strategies for identifying CD in children presenting with MIH as a sentinel manifestation.

This pilot case–control study aimed to assess the prevalence and severity of MIH in children with celiac disease, evaluate associated biochemical and intestinal permeability markers, and preliminary explore their possible relevance in identifying children with MIH who may require further assessment for CD.

## 2 Materials and methods

### 2.1 Study design and setting

A prospective pilot case–control study was conducted at the Department of Oral Pathology and the First Paediatric Clinic of “Louis Țurcanu” Emergency Clinical Hospital for Children over a 12-month period from 01 January 2025 to 31 December 2025. The study was approved by the Institutional Ethics Committee and was conducted in accordance with the Declaration of Helsinki. Prior to enrolment, written informed consent was obtained from the parents or legal guardians, and assent was obtained from the child when applicable.

### 2.2 Study population and eligibility criteria

A total of 70 children were enrolled: 35 with a confirmed diagnosis of celiac disease (CD group) and 35 age- and sex-matched healthy children (control group). Diagnosis of CD was established according to the European Society for Paediatric Gastroenterology, Hepatology and Nutrition (ESPGHAN) 2020 criteria, requiring elevated serum IgA anti-tissue transglutaminase antibodies (anti-tTG IgA ≥ 10 × upper limit of normal), positive anti-endomysial antibodies (EMA) or duodenal biopsy confirmation of villous atrophy (Marsh grade ≥2), combined with compatible clinical features (1).

Inclusion criteria: age 1–17 years, and absence of chronic systemic disease other than CD in the study group. Exclusion criteria: prior antibiotic therapy lasting >2 weeks in the first year of life (to exclude drug-induced MIH) (19), fluorosis, localised enamel defects of traumatic or infectious origin, ongoing orthodontic treatment, and incomplete laboratory data. The control group consisted of apparently healthy children attending routine paediatric reevaluations who were subsequently referred for dental examination. Children with known systemic diseases, developmental disorders, significant perinatal complications, chronic medication use, or other risk factors associated with enamel hypomineralisation were excluded.

Molar incisor hypomineralisation was diagnosed according to the criteria proposed by the European Academy of Paediatric Dentistry (EAPD) and the definition by Weerheijm et al. (20). MIH was defined as a qualitative enamel defect of systemic origin affecting one or more first permanent molars, with or without involvement of permanent incisors, presenting as demarcated opacities with altered enamel translucency. Lesions were distinguished from non-specific enamel defects, including diffuse opacities, enamel hypoplasia, fluorosis, and post-eruptive enamel breakdown of non-developmental origin. Only demarcated opacities affecting at least one first permanent molar were classified as MIH. Cases not fulfilling these criteria were recorded as non-specific enamel defects and were not included in MIH classification. Severity was recorded based on clinical criteria as described by Ghanim et al. (21), ranging from mild (demarcated opacities without enamel breakdown) to severe (post-eruptive enamel breakdown, atypical restorations, or extraction due to MIH-related structural failure).

### 2.3 Dental examination

Dental assessments were carried out by a single calibrated paediatric dentist under standardised conditions (artificial illumination, dental mirrors and compressed air tooth surface). Prior to the study, the examiner underwent calibration using reference photographs illustrating different grades of enamel defects according to the Aine classification. The intra-examiner reliability was assessed by re-examining 10% of participants 2 weeks later, yielding a Cohen’s kappa of 0.89.

**Figure 1:**
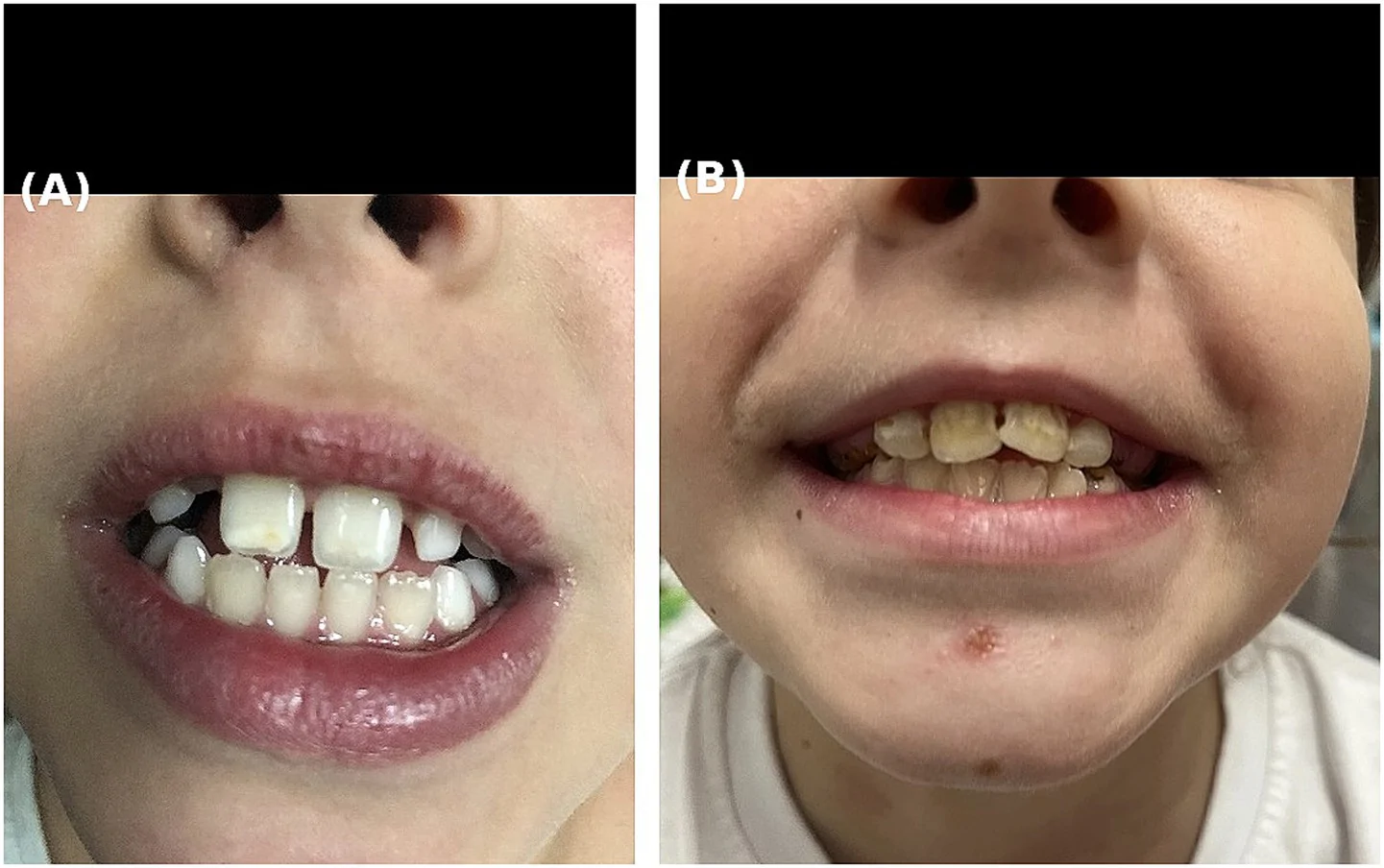
Clinical presentation of molar incisor hypomineralisation. **(A)** Mild MIH with demarcated creamy-white opacities on the maxillary central incisors. **(B)** Severe MIH showing yellow-brown opacities and post-eruptive enamel breakdown (PEB) on the incisal edges.

Severity was recorded based on clinical criteria adapted from Ghanim et al. (21), and further classified into a 0–4 ordinal scale (0 = no MIH; 1 = MIH without hypersensitivity and without defect; 2 = MIH without hypersensitivity but with defect; 3 = MIH with hypersensitivity but without defect; 4 = MIH with hypersensitivity and with defect), as shown in Figure 1. Enamel defect extent was graded using the Aine classification (4): 0 = no defects; 1 = minor changes in the enamel’s translucency; 2 = opacities or areas where the enamel’s is thinner than normal; 3 = hypoplasia more than 2/3 tooth’s surface; 4 = areas where the enamel is missing. Oral hygiene was assessed with the Löe Plaque Index (22), which evaluates the thickness of dental plaque at the gingival margin, specifically on the cervical third of teeth; score 0-no plaque in the area adjacent to the gingiva; score 1- a thin film of plaque detected by running a probe across the tooth surface; score 2- a moderate amount of soft deposits visible to the naked eye within the gingival pocket or on the gingival margin; score 3- heavy accumulation of soft matter (dense plaque) within the gingival pocket and/or on the gingival margin, and the Simplified Oral Hygiene Index (23): is a tool for rapid, quantitative assessment of oral hygiene, evaluating soft debris and calculus on six specific teeth, providing a total score (0 to 6) to rank hygiene as good, fair, or poor; scoring criteria (0–3): 0: no debris/calculus present;1: soft debris/calculus covering ≤13 of the tooth surface, or extrinsic stains; 2: soft debris/calculus covering >13 but ≤23 of the surface; 3: soft debris/calculus covering >23 of the surface.

### 2.4 Laboratory investigations

Fasting venous blood samples were collected from all participants. In the morning (minimum 8 h fasting) the following parameters were measured: serum vitamin K2 (menaquinone-7; High Performance Liquid Chromatography-HPLC), serum vitamin K1 (phylloquinone; High Performance Liquid Chromatography-HPLC), serum 25-hydroxyvitamin D-25(OH)D; [chemiluminescence immunoassay], serum zonulin (Enzyme Immunoassay (EIA)), serum iron (colorimetric), total calcium, phosphorus, alkaline phosphatase (ALP), alanine aminotransferase (GPT), and aspartate aminotransferase (GOT). Laboratory reference ranges were applied according to paediatric norms.

### 2.5 Statistical analysis

Statistical analyses were performed using IBM SPSS Statistics v26.0 (IBM Corp., Armonk, NY, United States). Normality of data distribution was assessed using the Shapiro–Wilk test. Since most variables exhibited non-normal distributions, non-parametric tests were used throughout. Between-group comparisons were performed with the Mann–Whitney U test for continuous variables and the χ^2^ test with Fisher’s exact correction for categorical variables. The Kruskal–Wallis test was used to compare MIH scores across age groups. Spearman’s rank correlation coefficient (r_s_) was used to quantify associations between continuous variables. Prior to logistic regression, multicollinearity was assessed using Variance Inflation Factor (VIF) and Tolerance statistics via an auxiliary linear regression. All predictors showed acceptable collinearity (VIF < 5; Tolerance > 0.20). Model goodness-of-fit was evaluated using the Hosmer–Lemeshow test. Variable selection followed the Enter method, with all three predictors (vitamin K2, vitamin D, and zonulin) entered simultaneously based on *a priori* biological plausibility and significant univariate associations with CD status (all *p* < 0.001). Model diagnostics included Cook’s distance and standardised residuals to identify influential observations. Binary logistic regression with 5,000 bootstrap resamples was performed to identify independent predictors of CD status, using vitamin K2, vitamin D, and zonulin as covariates. Receiver operating characteristic (ROC) curves and the area under the curve (AUC) were computed for each predictor and for the composite model. Statistical significance was set at *α* = 0.05. Sample size was calculated using G*Power 3.1 (24), targeting 80% power at α = 0.05 for a two-sided Mann–Whitney U test, yielding a minimum required *n* = 30 per group.

## 3 Results

### 3.1 Demographic characteristics

The two groups were well matched for age (CD median 6.67 years vs. control 6.92 years; Mann–Whitney U = 611.5, *p* = 0.995), sex (13 female/22 male in each group; χ^2^ = 0.00, *p* = 1.000), and dentition type (primary 42.9%, mixed 40.0%, permanent 17.1% in both groups). The demographic profile of the study population is summarised in Table 1.

**Table 1:** Demographic and clinical characteristics of the study population.

Characteristic	CD group (*n* = 35)	Control group (*n* = 35)	Statistic	*p*-value
Age, years – median (IQR)	6.67 (3.50–11.08)	6.92 (3.50–11.00)	U = 611.5	0.995
Sex, Female/Male – *n* (%)	13/22 (37.1/62.9)	13/22 (37.1/62.9)	χ^2^ = 0.00	1.000
Dentition – *n* (%)				
Primary	15 (42.9)	15 (42.9)	–	–
Mixed	14 (40.0)	14 (40.0)	–	–
Permanent	6 (17.1)	6 (17.1)	–	–
MIH prevalence – *n* (%)	**27 (77.1%)**	**0 (0.0%)**	**OR = ∞; Fisher**	**<0.001**

IQR, interquartile range; U, Mann–Whitney U statistic; χ^2^, chi-square statistic; OR, odds ratio. *p*-values were calculated using the Mann–Whitney U test, chi-square test, or Fisher’s exact test, as appropriate. Bold values indicate statistical significance (*p* < 0.05).

**Figure 2:**
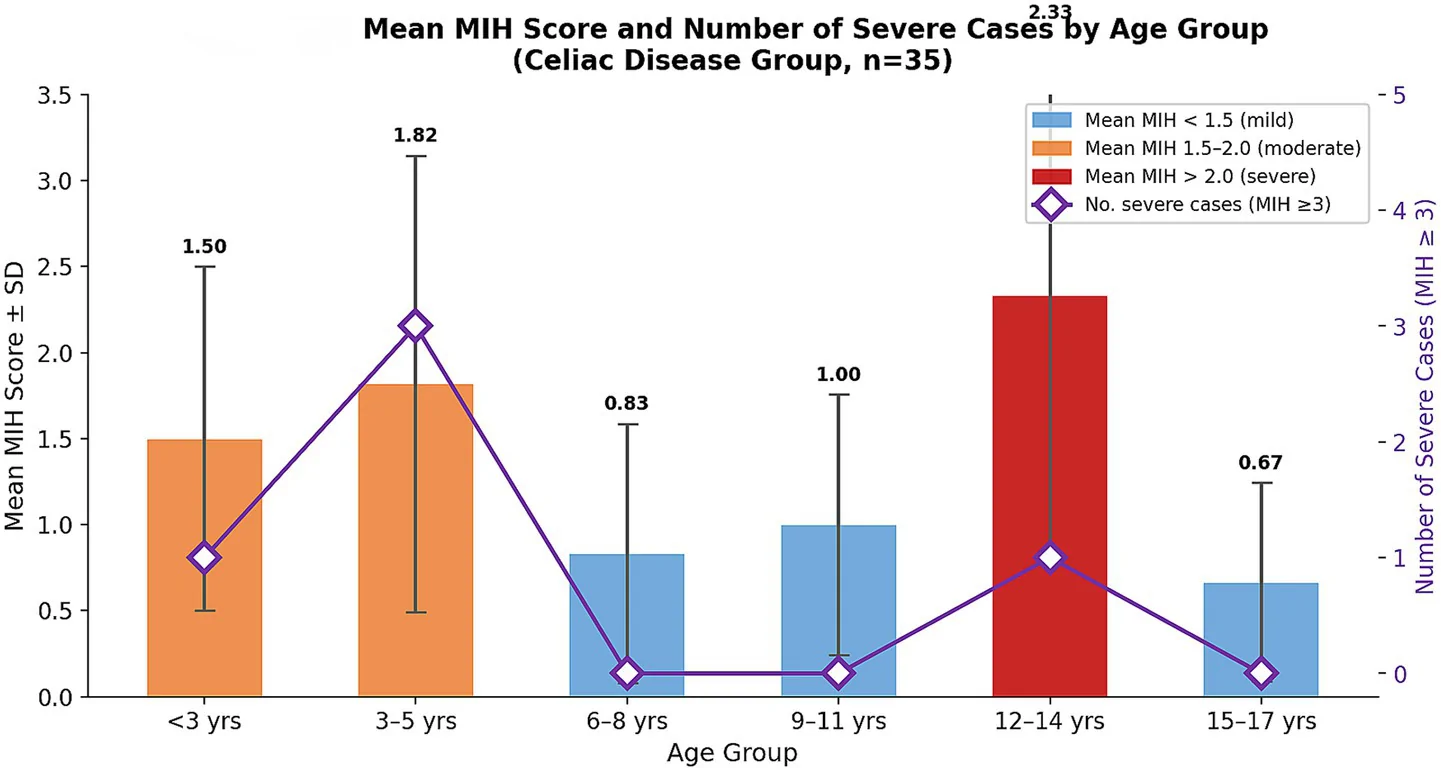
Mean MIH score and number of severe cases by age group in the celiac disease group. Bar chart of mean MIH score ± standard deviation per age group (left y-axis), colour-coded by severity level (blue = mean <1.5; orange = mean 1.5–2.0; red = mean >2.0). The purple diamond-line overlay (right y-axis) indicates the absolute count of severe MIH cases (score ≥3) per age group. The 3–5 year group carries the highest absolute burden of severe cases (*n* = 3), while the 12–14 year group shows the highest mean score. The 6–8, 9–11, and 15–17 year groups contain no severe cases.

Age-stratified analysis of MIH severity within the celiac disease group revealed a non-uniform distribution of defect burden across developmental stages. The highest mean MIH score was recorded in the 12–14 year age group (mean 2.33 ± 1.53), driven by a single case requiring tooth extraction (MIH score 4). However, the 3–5 year group carried the greatest absolute number of severe cases (MIH score ≥3; *n* = 3), representing 27.3% of children in that cohort, followed by the <3 year group (mean MIH 1.50 ± 1.29; one severe case). In contrast, the 6–8, 9–11, and 15–17 year groups contained no cases with MIH score ≥3, displaying predominantly milder defects (score 1). Kruskal-Wallis test did not reveal a statistically significant difference in MIH scores across age groups (H = 6.833, *p* = 0.233), likely reflecting the limited size of individual subgroups; findings are therefore interpreted descriptively (Figure 2).

### 3.2 Prevalence and severity of MIH and enamel defects

MIH was present in 27 of 35 children with CD (77.1%) compared with none of the 35 controls (0.0%), a difference that was highly statistically significant (Fisher *p* < 0.001, OR = ∞). In the CD group, MIH severity scores ranged from 1 to 4, with score 1 being the most prevalent (*n* = 8). Notably, scores 3 and 4 were observed exclusively observed in this group.

**Figure 3:**
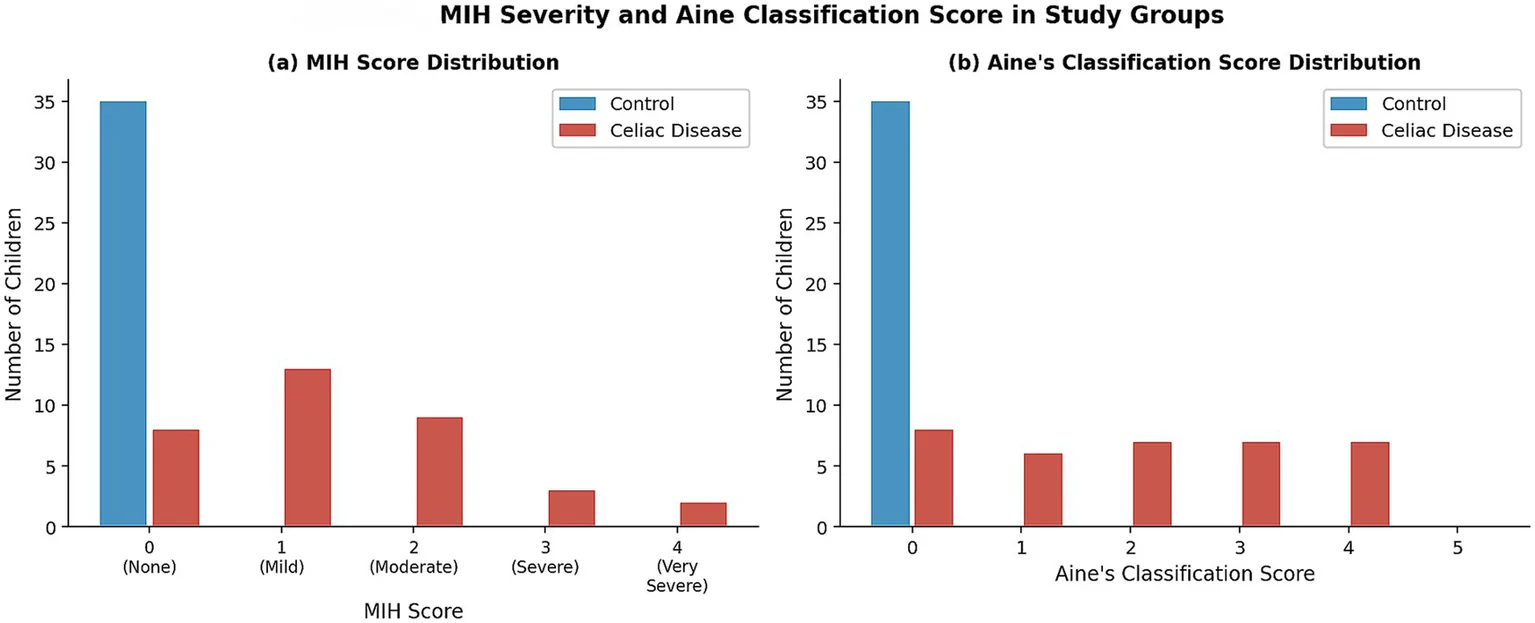
Distribution of MIH severity scores and Aine classification scores. **(a)** Bar chart of MIH severity scores (0–4) across celiac disease and control groups. All MIH-positive scores are confined to the CD group. **(b)** Bar chart of Aine’s enamel classification scores, confirming severe enamel involvement exclusively in CD children. CD, celiac disease.

Similarly, the Aine classification score was significantly higher in children with celiac disease (median 2.00, IQR 0.00–3.00) than in controls (median 0.00, IQR 0.00–0.00; U = 1085.0, *p* < 0.001). The distribution of MIH severity and Aine classification scores in both groups is shown in Figure 3.

### 3.3 Fat-soluble vitamins and biochemical markers

Serum vitamin K2 was significantly lower in children with celiac disease (median 0.340 μg/L, IQR 0.24–0.46) than in controls (0.637 μg/L, IQR 0.56–0.74; U = 68.0, *p* < 0.001). Similarly, serum vitamin D was markedly lower in the CD group (median 24.10 ng/mL) versus controls (43.10 ng/mL; U = 94.0, *p* < 0.001). Although serum vitamin K1 was also significantly reduced in CD (0.100 vs. 0.820 μg/L; *p* < 0.001), its correlation with MIH severity did not reach significance (r_s_ = 0.000, *p* = 0.998), suggesting the association may be predominantly mediated through vitamin K2 and vitamin D.

Serum calcium (8.6 vs. 9.8 mg/dL; *p* < 0.001), phosphorus (3.4 vs. 4.6 mg/dL; *p* < 0.001), and iron (62.0 vs. 115.0 μg/dL; *p* < 0.001) were all substantially lower in CD children, consistent with the pan-absorptive dysfunction characteristic of active mucosal disease. Zonulin, a biomarker of intestinal permeability, was markedly elevated in CD (median 72.10 ng/mL) compared with controls (21.20 ng/mL; U = 1225.0, *p* < 0.001). Liver enzymes (GPT, GOT) and ALP were elevated in CD, reflecting subclinical hepatobiliary involvement and increased bone turnover. Full results are presented in Table 2.

**Table 2:** Comparison of serum biomarkers between the celiac disease and control groups.

Variable	Celiac disease median (IQR)	Control median (IQR)	Mann–Whitney U	*p*-value
Vitamin K2 (μg/L)	0.340 (0.24–0.46)	0.637 (0.56–0.74)	68.0	**<0.001**
Vitamin D (ng/mL)	24.10 (17.5–30.6)	43.10 (37.8–47.3)	94.0	**<0.001**
Vitamin K1 (μg/L)	0.100 (0.06–0.36)	0.820 (0.61–0.93)	119.0	**<0.001**
Zonulin (ng/mL)	72.10 (48.0–93.9)	21.20 (17.9–24.7)	1225.0	**<0.001**
Serum Iron (μg/dL)	62.00 (42.5–80.0)	115.0 (105–120)	33.0	**<0.001**
Calcium (mg/dL)	8.60 (8.3–9.1)	9.80 (9.6–10.0)	55.0	**<0.001**
Phosphorus (mg/dL)	3.40 (3.1–3.9)	4.60 (4.4–4.8)	40.0	**<0.001**
ALP (U/L)	235 (188–280)	148 (135–164)	1280.0	**<0.003**
GPT (U/L)	35.0 (24.0–49.0)	16.0 (13.0–21.0)	1141.0	**<0.002**
GOT (U/L)	33.0 (23.0–45.0)	15.0 (13.0–20.0)	1310.0	**<0.004**

IQR, interquartile range; U, Mann–Whitney U statistic. *p*-values were calculated using the Mann–Whitney U test and adjusted for multiple comparisons using the Bonferroni method where applicable. Bold values indicate statistically significant differences between groups (*p* < 0.05).

**Figure 4:**
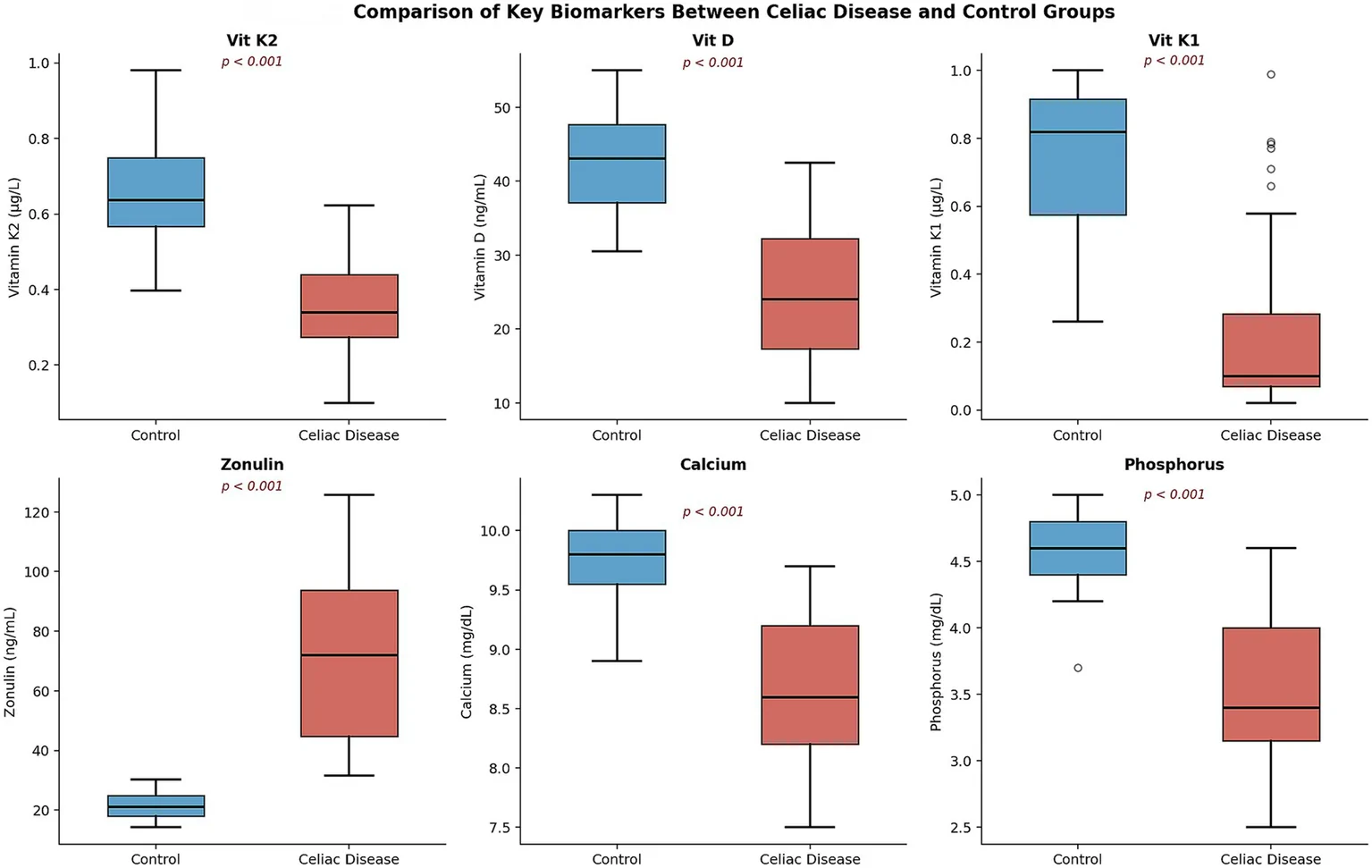
Comparison of key serum biomarkers between study groups. Box plots showing median, interquartile range, and outliers for vitamin K2, vitamin D, vitamin K1, zonulin, calcium, and phosphorus in celiac disease (red) and control (blue) groups. All comparisons are statistically significant (*p* < 0.001 by Mann–Whitney U test).

Figure 4 is more representative for the difference of this parameters in both groups.

**Figure 5:**
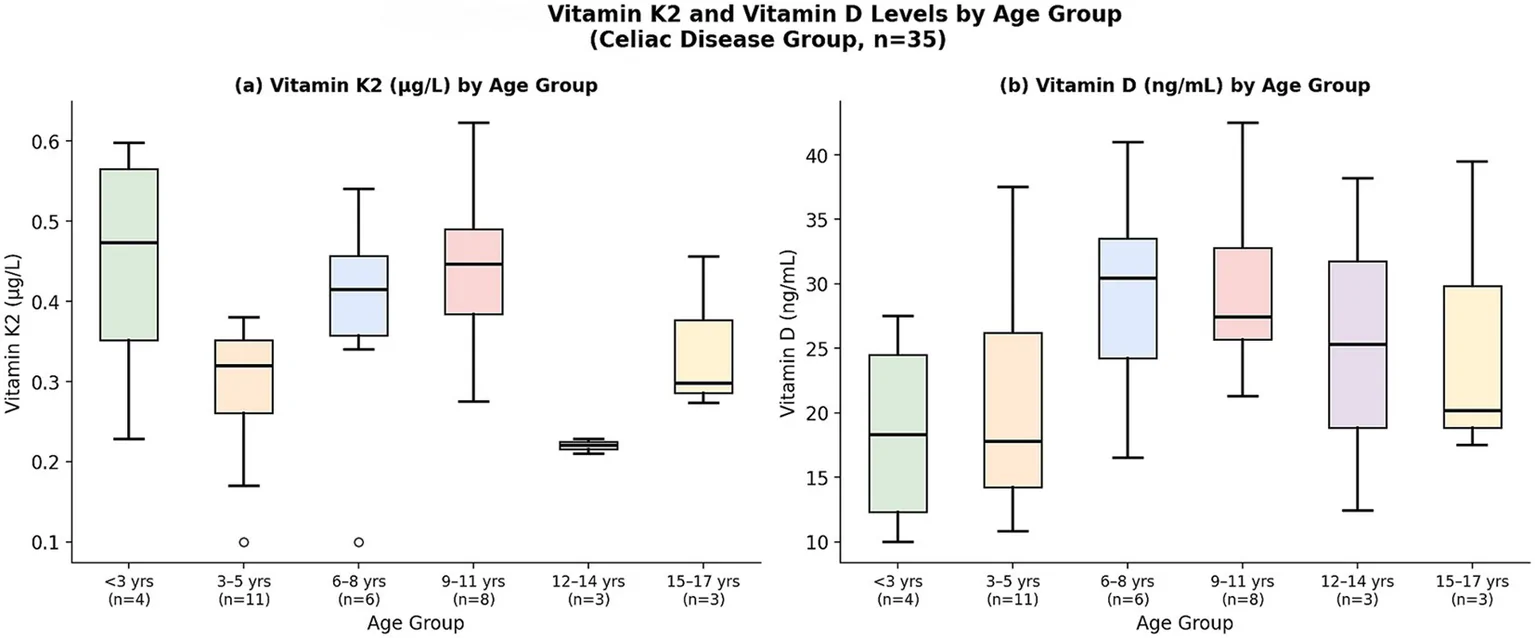
Vitamin K2 and vitamin D levels stratified by age group in the celiac disease group. **(a)** Box plots of serum vitamin K2 (μg/L) by age group. **(b)** Box plots of serum vitamin D [25(OH)D, ng/mL] by age group. The horizontal dashed line represents vitamin D sufficiency threshold (20 ng/mL). Youngest age groups (<3 and 3–5 years) display the lowest median vitamin D concentrations, consistent with the clinical observation that these groups bear the highest MIH severity burden. Each box shows median (central line), IQR (box limits), and range (whiskers); individual outliers shown as circles.

Stratification of fat-soluble vitamin levels by age group revealed that the youngest cohorts exhibited the most severe nutritional depletion. Median serum vitamin D concentrations were lowest in the <3 year group (18.3 ng/mL) and the 3–5 year group (17.8 ng/mL), both below the broadly accepted paediatric sufficiency threshold of 20 ng/mL, compared with higher median levels in the 6–8 year (30.4 ng/mL) and 9–11 year groups (27.5 ng/mL). Serum vitamin K2 followed a similar pattern, with the 3–5 year group recording the lowest median value (0.320 μg/L), well below the paediatric reference range. Notably, the 12–14 year group displayed a markedly reduced median vitamin K2 (0.221 μg/L), the lowest across all groups, corresponding to the highest mean MIH score in the cohort. The concordance between the age groups with the most depleted vitamin K2 and D levels and those bearing the greatest MIH severity burden provides additional support for the mechanistic role of fat-soluble vitamin deficiency in enamel mineralisation failure during celiac disease (Figure 5).

### 3.4 Oral hygiene indices

Oral hygiene indicators were significantly poorer in the CD group. The Löe Plaque Index was higher in children with celiac disease (median 0.70 vs. 0.30; U = 1055.0, *p* < 0.001). The OHI-S score was also significantly higher in the CD group (median 1.20 vs. 0.50; U = 1129.0, *p* < 0.001) as shown in Table 3.

**Table 3:** Oral health indices: MIH severity, enamel classification, and hygiene scores.

Oral Health Index	Celiac disease median (IQR)	Control median (IQR)	Mann–Whitney U	*p*-value
MIH score	1.00 (0.00–2.00)	0.00 (0.00–0.00)	1085.0	**<0.001**
Aine classification score	2.00 (0.00–3.00)	0.00 (0.00–0.00)	1085.0	**<0.001**
Löe Plaque Index	0.70 (0.60–1.00)	0.30 (0.20–0.50)	1055.0	**<0.001**
OHI-S score	1.20 (0.90–1.40)	0.50 (0.40–0.80)	1129.0	**<0.001**

Values are medians (IQR). U, Mann–Whitney U statistic; OHI-S, Simplified Oral Hygiene Index. Bold values indicate statistically significant differences between groups (*p* < 0.05).

### 3.5 Correlation analysis

**Figure 6:**
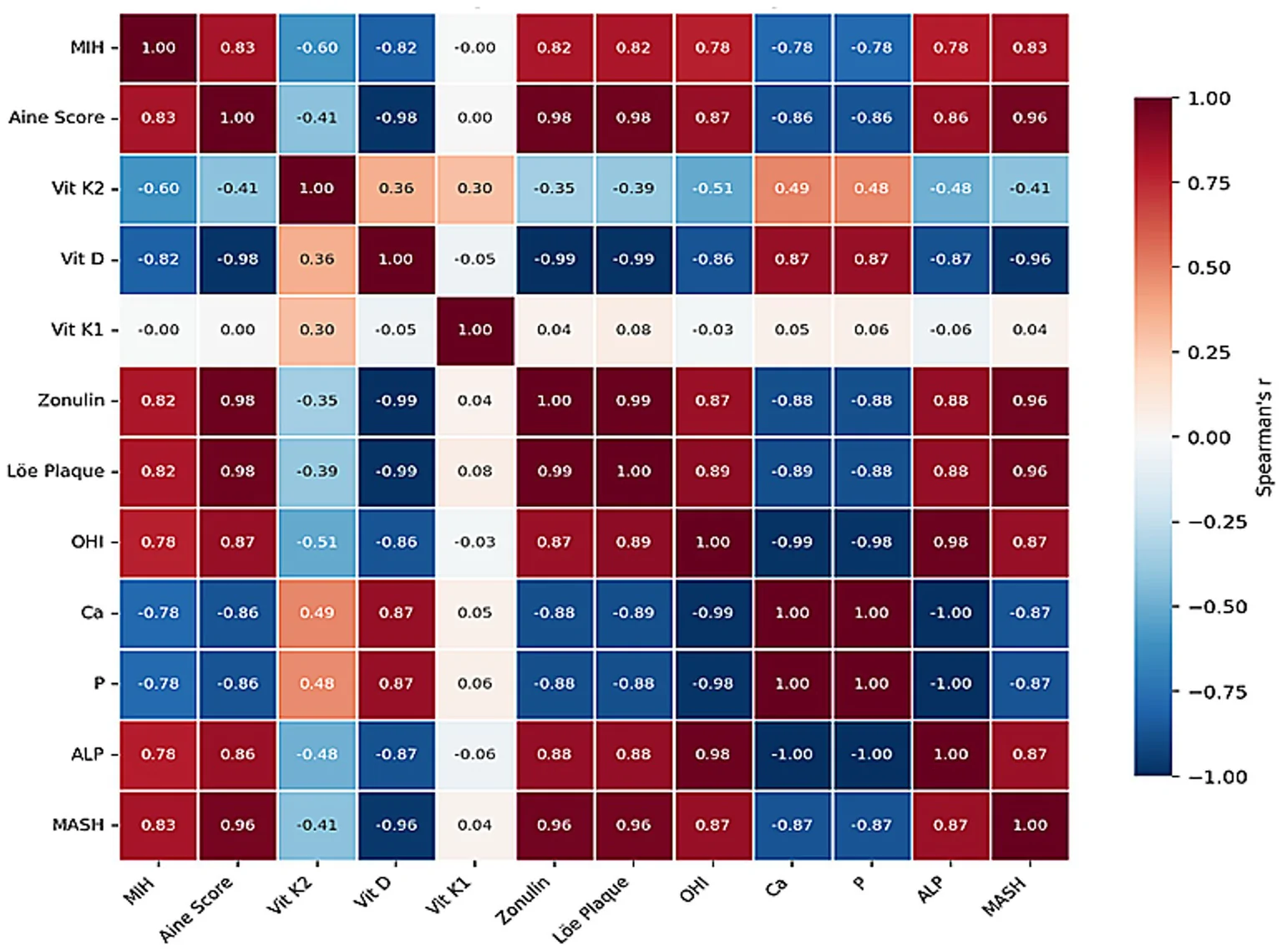
Spearman correlation matrix for the celiac disease group. Heat map of Spearman’s *rs* values between MIH, Aine’s score, and biochemical/oral-health variables in the celiac disease group (*n* = 35). Red shades indicate positive correlations; blue shades indicate negative correlations. All correlations with |*rs*| > 0.40 are statistically significant (*p* < 0.05).

Within the CD group, MIH severity demonstrated strong statistically significant correlations with all major biochemical and clinical parameters (Figure 6). The strongest negative correlations were observed with vitamin D (r_s_ = −0.818, *p* < 0.001) and serum iron (r_s_ = −0.817, *p* < 0.001), followed by vitamin K2 (r_s_ = −0.597, *p* < 0.001) and serum calcium (rs = −0.782, *p* < 0.001). Strong positive correlations were found between MIH severity and zonulin (rs = +0.818), Aine score (r_s_ = +0.831) and Löe Plaque Index (r_s_ = +0.818).

Notably, the Aine classification score showed an almost perfect negative correlation with vitamin D (r_s_ = −0.980, *p* < 0.001) and a near-perfect positive correlation with zonulin (r_s_ = +0.980, *p* < 0.001), indicating that the extent of enamel defect involvement closely tracks the degree of intestinal permeability and vitamin D depletion. In the full dataset (*n* = 70), vitamin K2 demonstrated a strong negative correlation with MIH severity (r_s_ = −0.748, *p* < 0.001) and Aine score (r_s_ = −0.684, *p* < 0.001), highlighting its clinical relevance across the entire sample.

**Figure 7:**
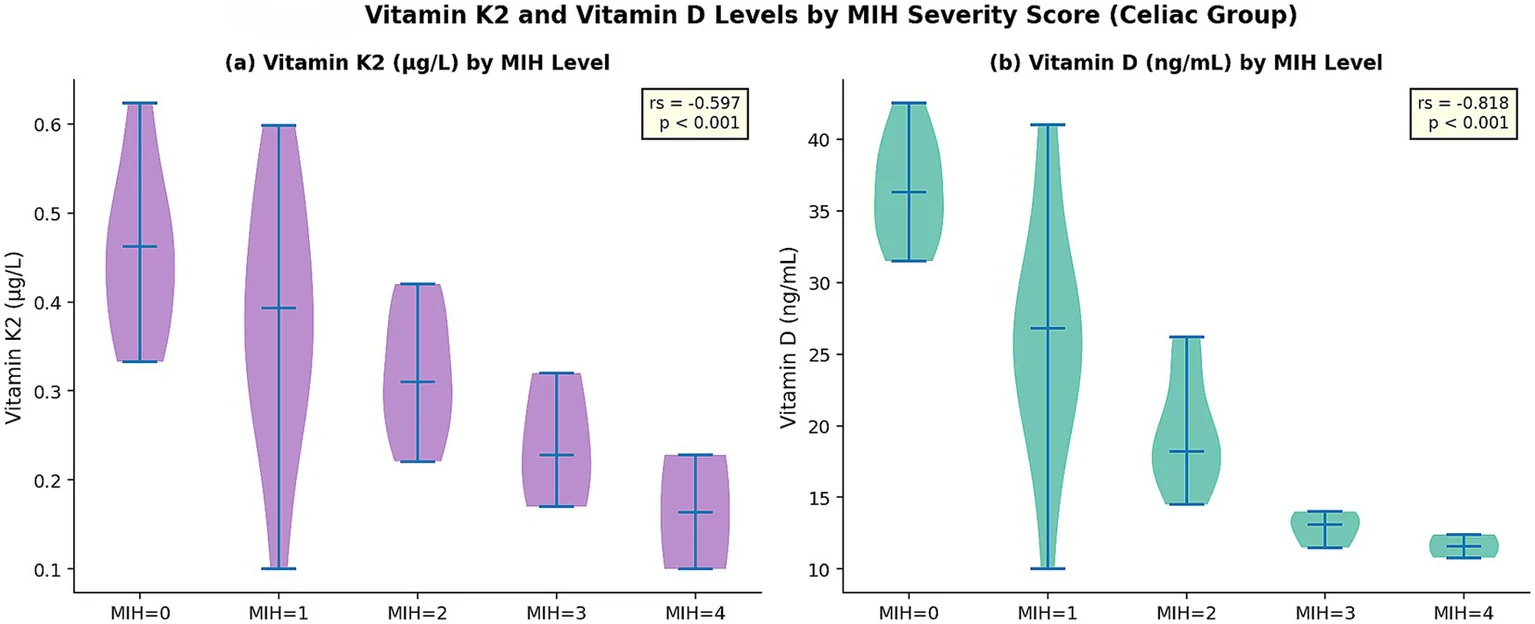
Distribution of vitamin K2 and vitamin D levels by MIH severity score in the celiac disease group. **(a)** Violin plots showing serum vitamin K2 levels stratified by MIH score (0–4) in the CD group. **(b)** Corresponding vitamin D distributions by MIH score. A progressive decline in both vitamins is observed with increasing MIH severity.

The relationship between nutritional level of vitamins K2 and D and dental health was further explored using Spearman correlation analysis. The results demonstrated a significant inverse association between MIH severity and serum levels of fat-soluble vitamins: Vitamin D showed a very strong negative correlation (r_s_ = −0.818, *p* < 0.001), indicating that MIH severity increases from 0 to 4 as vitamin D levels decrease. Similarly, vitamin K2 showed a moderate-to-strong negative correlation (r_s_ = −0.597, *p* < 0.001), indicating that lower concentrations are associated with more extensive enamel hypomineralisation, as shown in Figure 7.

### 3.6 Diagnostic performance – logistic regression and ROC analysis

Multivariable logistic regression analysis identified vitamin D and zonulin as significant independent predictors of celiac disease (*p* < 0.001), with associations confirmed using bootstrap confidence intervals. Vitamin K2 showed a negative association; however, this did not reach statistical significance (*p* = 0.089). Overall, the model indicates a strong relationship between zonulin levels, vitamin D status, and celiac disease risk, as seen in Table 4.

**Table 4:** Multivariable binary logistic regression analysis of factors associated with celiac disease.

Predictor	B	Boot SE	95%CI	*p*-value
Vitamin D	2.413	0.478	0.743, 2.734	**<0.001**
Vitamin K2	−37.990	21.586	−98.302, −4.163	0.089
Zonulin	3.575	0.385	2.249, 3.743	**<0.001**

B, regression coefficient; Boot SE, bootstrap standard error; CI, confidence interval. The model was estimated using multivariable binary logistic regression with 5,000 bootstrap resamples. *p*-values refer to Wald tests. Bold values indicate statistically significant differences between groups (*p* < 0.05).

**Figure 8:**
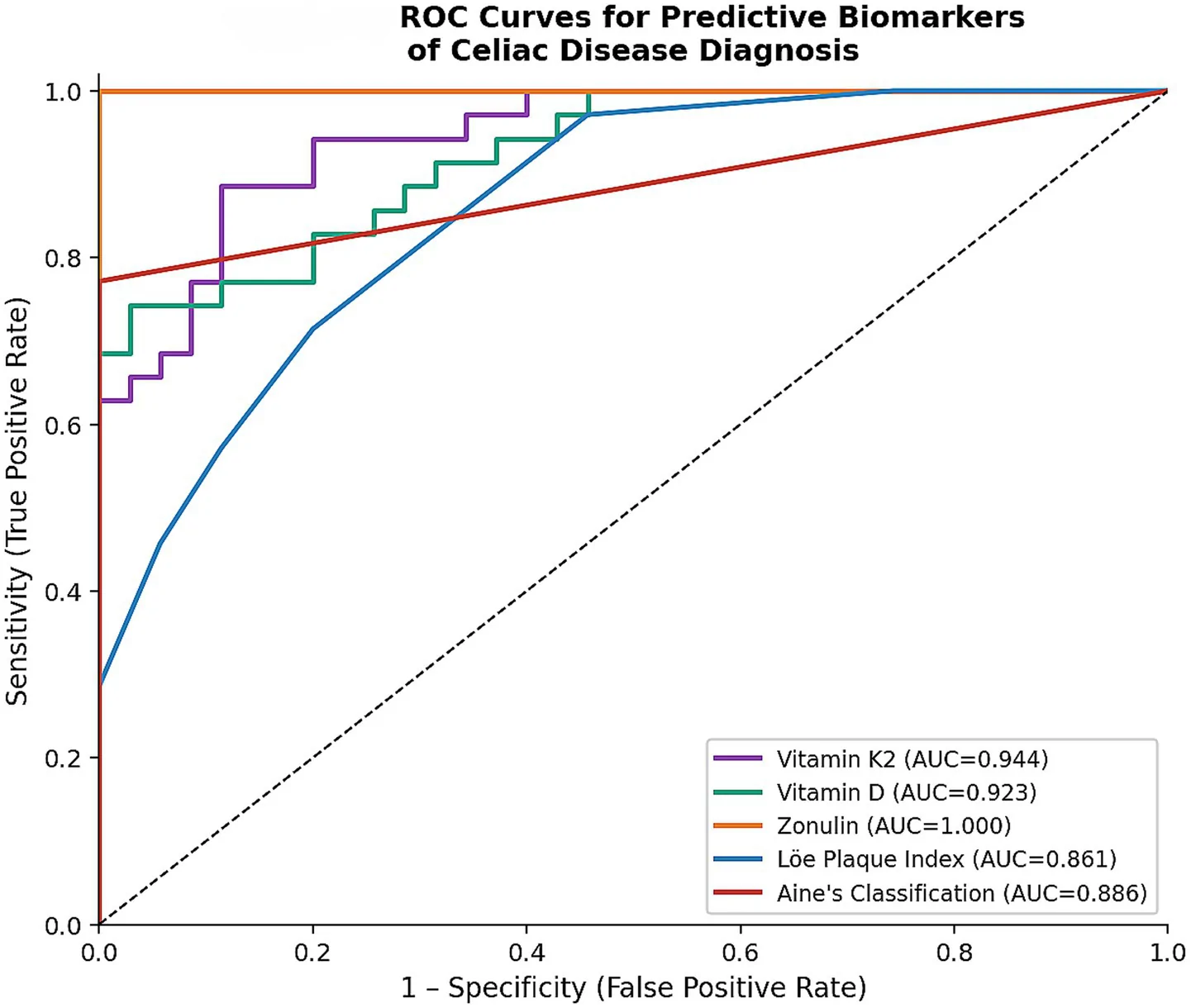
ROC curves for individual biomarkers in the discrimination of celiac disease. Receiver operating characteristic (ROC) curves for zonulin, vitamin K2, vitamin D, Aine classification score, and the Löe Plaque Index in identifying celiac disease status. AUC values are provided in the legend.

ROC analysis demonstrated excellent discriminatory performance of the evaluated biomarkers in distinguishing children with celiac disease from controls. Zonulin achieved an AUC of 1.000, followed by vitamin K2 (AUC = 0.944, 95% CI 0.887–0,999) and vitamin D (AUC = 0.923, 95% CI 0.857–0.989). Both the Aine classification and MIH score yielded an AUC of 0.886 (95% CI 0.799–0.972), while the Löe Plaque Index achieved an AUC of 0.861 (95% CI 0.778–0.944), as shown in Figure 8.

## 4 Discussion

Celiac disease involves complex enteric alterations that lead to severe malabsorption phenomena. Current diagnosis benefits from the updated guidelines of the ESPGHAN, which standardise the diagnostic approach and assess both local and systemic changes induced by celiac disease. However, the oral cavity remains insufficiently explored in the context of systemic paediatric pathology.

Dental and oral changes associated with celiac disease are well documented, including an increased prevalence of dental caries, oral aphthae, while the association with molar–incisor hypomineralization remains insufficiently characterised.

The relationship between fat-soluble vitamins D and K and both dental and bone mineralisation is well established. Given the relatively high prevalence of MIH observed in patients with celiac disease, we hypothesised a possible association with deficiencies in these vitamins.

The principal finding of this study is the high prevalence of MIH in children with celiac disease, (77.1%) compared with no cases in age- and sex-matched healthy controls. This exceeds population estimates (14.2%) reported in systematic reviews (25), although comparisons should be interpreted cautiously due to differences in populations and study design. These findings are consistent with previous reports suggesting an association between celiac disease and enamel developmental disturbances (3, 26). The absence of MIH in the control group may be related to strict inclusion criteria applied for healthy participants, which excluded children with systemic disease, premature birth or medical conditions known to affect enamel development. The observed findings are hypothesis-generating and suggest a possible association between early childhood exposure to celiac disease-related metabolic disturbances and molar-incisor hypomineralisation. Severe MIH (scores 3–4) was most frequently observed in younger children (2.7–5 years) at the time of examination, which may reflect variability in MIH expression across developmental stages. The higher mean MIH score observed in the 12–14-year age group was driven by a single outlier (ID 4, age 13.7 years) with MIH score 4 and marked vitamin D deficiency (12.4 ng/mL). Given the small sample size, this observation should be interpreted cautiously, as individual cases may disproportionately affect group-level estimates. The absence of severe MIH in the 6–8 and 9–11-year age groups, despite a relatively high overall prevalence of MIH (50–75%), may reflect variability in MIH expression across individuals and developmental stages. Differences in tooth eruption timing and the clinical detectability of enamel defects at the time of examination may also have contributed to these findings. This age-stratified analysis of MIH severity in children with celiac disease showed that the youngest age groups (<3 and 3–5 years) had lower median vitamin D levels and higher Aine scores at the time of examination. These findings may suggest an association between systemic metabolic disturbances and enamel defect severity; however, no causal inference can be made due to the cross-sectional design. It should also be considered that the enamel mineralisation of first permanent molars occurs during early childhood, whereas biochemical parameters were measured at the time of study enrollment. Therefore, the present findings cannot establish a temporal relationship but may reflect persistent systemic alterations associated with this disease.

Vitamin K2 is the cofactor for carboxylation of two proteins critical to enamel and dentine mineralisation: osteocalcin and matrix Gla protein (MGP) (16, 27). Undercarboxylated osteocalcin, as a consequence of vitamin K2 insufficiency, impairs calcium binding in the organic matrix of developing enamel, yielding structurally deficient mineralisation zones that present clinically as hypomineralised opacities. A significant inverse correlation was observed between MIH severity and vitamin K2 levels (r_s_ = −0.597, *p* < 0.001). Our data provide the first direct quantitative evidence linking serum menaquinone deficiency to MIH severity in children with celiac disease.

Vitamin D regulates the transcription of calbindin-D9K and TRPV5/6 calcium channels in ameloblasts, a process indispensable for the active calcium flux required during the maturation stage of amelogenesis (28, 29). In our study, vitamin D deficiency was the variable most strongly correlated with both MIH severity (r_s_ = −0.818, *p* < 0.001) and the Aine classification score (r_s_ = −0.980, *p* < 0.001). The synergy between vitamin D and vitamin K2 in mineralised tissue formation has been conceptualised as a “vitamins D-K2 interaction axis,” in which vitamin D promotes calcium absorption, while vitamin K2 directs calcium to sites of mineralisation and inhibits soft tissue calcification (17). The present findings are consistent with this model in children with celiac disease. It should also be considered that the enamel mineralisation of first permanent molars occurs during early childhood, whereas biochemical parameters were measured at the time of study enrollment. Therefore, the present findings cannot establish a temporal relationship but may reflect persistent systemic alterations associated with this disease.

An interesting observation in this study was the strong association between the severity of enamel defects and biomarkers related to intestinal permeability and micronutrient status. This finding suggests that enamel defects may reflect underlying systemic alterations associated with celiac disease. If confirmed in larger studies, dental examination could serve as a useful clinical indicator of systemic disturbances in paediatric celiac disease. Intestinal barrier dysfunction can be assessed using biomarkers such as zonulin, which regulates tight junctions and increases paracellular permeability, facilitating the passage of dietary antigens and contributing to autoimmune process perpetuation (6).

A strong correlation was observed between the Aine classification score and both zonulin (rₛ = 0.980) and vitamin D levels (rₛ = −0.980). While these coefficients suggest a relationship between enamel defect severity, intestinal permeability, and vitamin D status in children with celiac disease, correlations of this magnitude are unusual in biological studies and must be interpreted cautiously. The use of an ordinal metric, such as the Aine classification, in a small and homogeneous pilot cohort can artificially inflate correlation values, particularly if the data are clustered or lack substantial variability across categories. Therefore, these extreme coefficients (≈ ± 0.98) likely reflect sample characteristics rather than a true perfect association, making these findings hypothesis-generating rather than confirmatory.

Nevertheless, despite these methodological limitations, the underlying trends suggest a clinically relevant concept. Although zonulin testing is not universally available in primary care, combining dental enamel assessments with routine vitamin D screening could offer a non-invasive and cost-effective approach for evaluating children with suspected systemic conditions. As emphasised by Theethira and Dennis, the early identification of celiac disease is essential to prevent the long-term complications of gluten exposure, including osteoporosis and lymphoma (30).

The finding that oral hygiene indices (Löe Plaque Index, OHI-S) were significantly poorer in children with celiac disease may be explained by MIH-related pain or discomfort, which can reduce the quality and regularity of tooth brushing. In addition, the structural fragility of hypomineralised enamel renders affected surfaces more susceptible to plaque retention and early carious destruction (21). Paediatric dentists encountering children with MIH-affected teeth may consider a dual approach, including intensive preventive management of dental lesions and referral for serological screening for celiac disease.

These findings may strengthen the clinical relevance of MIH, particularly in cases with severe or bilaterally symmetric enamel defects, as a potential oral manifestation associated with celiac disease. From a clinical perspective, they support the hypothesis that children presenting with MIH could benefit from further evaluation for underlying systemic conditions, including celiac disease, within a multidisciplinary diagnostic framework. In addition, the observed association between MIH severity and fat-soluble vitamin deficiencies raises the hypothesis that early nutritional optimisation, including vitamin D and K2 status correction after celiac disease diagnosis, may play a supportive role in systemic management; however, interventional studies are required to confirm any potential protective effect.

Several limitations of the present study should be acknowledged. The single-centre design and the 12-month recruitment period limit the generalizability of the findings. Although *a priori* a power analysis indicated adequacy for the primary endpoint (*n* = 30 per group required; 35 enrolled), the relatively small sample size may have increased the risk of overfitting and contributed to an overestimation of observed associations and diagnostic performance. In addition, it may have contributed to the absence of MIH cases in controls by chance. Regarding the analytical model, the composite logistic regression model was developed and tested within the same dataset without internal or external validation, limiting its generalizability. Validation in independent cohorts is required before clinical application.

The cross-sectional design further precludes causal or temporal inference regarding vitamin status, as measurements reflect current biochemical status rather than conditions during the critical period of enamel formation. In addition, dietary adherence to a gluten-free diet was not systematically recorded, meaning that biochemical markers may reflect current disease activity rather than long-term nutritional status. Potential residual confounding cannot be excluded, as socioeconomic status, oral hygiene behaviours beyond clinical indices, sun exposure, supplement use, genetic susceptibility (e.g., HLA-DQ2/DQ8 status), and degree of intestinal mucosal involvement were not systematically assessed.

Overall, these limitations should be considered when interpreting the findings, which are exploratory and hypothesis-generating.

## 5 Conclusion

This present study suggests a high prevalence of molar-incisor hypomineralisation amongst children with celiac disease compared with healthy controls, supporting a possible association between enamel defects and this systemic disorder. In addition, lower vitamin K2 and vitamin D levels were correlated with greater MIH severity, providing preliminary evidence for a potential link between fat-soluble vitamin deficiency and impaired enamel mineralisation in children with celiac disease. Given the limited sample size and single-center design, these findings should be interpreted cautiously and considered exploratory.

Our results indicate that zonulin, vitamin K2, and vitamin D showed promising discriminatory performance in distinguishing children with celiac disease from controls; however, these findings remain exploratory and require validation in larger cohorts before clinical interpretation. These observations further suggest a potential relevance of structured dental evaluation within the multidisciplinary care of children with celiac disease, although larger multicenter studies are needed before broader clinical implications can be established.

## Data Availability

The original contributions presented in the study are included in the article/supplementary material, further inquiries can be directed to the corresponding authors.
